# Syntheses, Spectral Characterization, and Antimicrobial Studies on the Coordination Compounds of Metal Ions with Schiff Base Containing Both Aliphatic and Aromatic Hydrazide Moieties

**DOI:** 10.1155/2013/981764

**Published:** 2013-10-03

**Authors:** Dinesh Kumar, Silky Chadda, Jyoti Sharma, Parveen Surain

**Affiliations:** ^1^Department of Chemistry, National Institute of Technology, Kurukshetra, Haryana 136119, India; ^2^Department of Microbiology, Kurukshetra University, Kurukshetra, Haryana 136119, India

## Abstract

An EtOH solution of 3-ketobutanehydrazide and salicylhydrazide on refluxing in equimolar ratio forms the corresponding Schiff base, LH_3_ (**1**). The latter reacts with Mn(II), Co(II), Ni(II), Cu(II), Zn(II), Cd(II), Zr(OH)_2_(IV), MoO_2_(VI), and UO_2_(VI) ions in equimolar ratio and forms the corresponding coordination compounds, [M(LH)(MeOH)_3_] (**2**, M = Mn, Co, Ni), [Cu(LH)]_2_ (**3**), [M′(LH)(MeOH)] (**4**, M′ = Zn, Cd), [Zr(OH)_2_(LH)(MeOH)_2_] (**5**), [MoO_2_(LH)(MeOH)] (**6**), and [UO_2_(LH)(MeOH)] (**7**). The coordination compounds have been characterized on the basis of elemental analyses, molar conductance, spectral (IR, reflectance, ^1^H NMR, ESR) studies, and magnetic susceptibility measurements. They are nonelectrolytes in DMSO. The coordination compounds, except **3**, are monomers in diphenyl. They are active against gram-positive bacteria (*S. aureus, B. subtilis*), gram-negative bacteria (*E. coli, P. aeruginosa*), and yeast (*S. cerevisiae, C. albicans*). **1** acts as a dibasic tridentate ONO donor ligand in **2–7** coordinating through its both enolic O and azomethine N atoms. The coordination compounds **2** and **3** are paramagnetic, while rest of the compounds are diamagnetic. A square-planar structure to **3**, a tetrahedral structure to **4**, an octahedral structure to **2**, **6**, and **7**, and a pentagonal bipyramidal structure to **5** are proposed.

## 1. Introduction

Aroyl hydrazones and their coordination compounds are known to possess the biological activities and inhibit many enzymatic reactions in the cell. Owing to their biological activities such as antifungal, antibacterial, antimycobacterial, antitumor, anti-inflammatory, anti-HIV, leishmanicidal, trypanocidal, inhibitor of anthrax lethal factor, antidiabetic, antimalarial, and antipyretic, there has been an increasing interest towards the studies of the coordination compounds of the Schiff bases containing the hydrazone moiety during the past few decades [1–12]. The coordination compounds containing hydrazone moiety have been reported to act as analytical reagents, such as polymer coatings, fluorescent materials [13, 14], enzymes inhibitors, antifungal/antibacterial agents [15, 16], and corrosion inhibitors [17]. A perusal of the literature reveals that much work has been carried out towards the coordination compounds of Schiff bases containing salicylhydrazide moiety [18–27]; however, no work seems to be reported on the coordination compounds of Schiff base derived from 3-ketobutanehydrazide and salicylhydrazide. Novel noncytotoxic salicylhydrazide-containing 1N inhibitors have been developed through substructure database search methods [28]. The developmental progress of the salicylhydrazide class of 1N inhibitors was halted due to cytotoxicity issues. The salicyloylhydrazide moiety has been reported to be the minimally required substructure for 1N inhibitory potency of the compounds [29]. The salicylhydrazides have also been proposed to inhibit 1N catalytic activity through chelation of the active site Mg^2+^, and they exhibit cytotoxicity in the nanomolar range. The replacement of one of the two phenols in N,N′-bis-salicylhydrazide with an optimally substituted heterocyclic group (heavily substituted triazole groups) renders a novel class of noncytotoxic salicylhydrazides, greatly enhancing the therapeutic potential of this class of 1N inhibitors. Keeping in view the above importance of the compounds possessing hydrazone moiety, we thought it worthwhile to synthesize and characterize the Schiff base, LH_3_ (**1**) and its coordination compounds with Mn(II), Co(II), Ni(II), Cu(II), Zn(II), Cd(II), Zr(OH)_2_(IV), MoO_2_(VI), and UO_2_(VI) ions. The Schiff base and its coordination compounds have also been studied for their antimicrobial activities.

## 2. Experimental

### 2.1. Materials

Manganese(II) acetate tetrahydrate, cobalt(II) acetate tetrahydrate, nickel(II) acetate tetrahydrate, copper(II) acetate monohydrate, ethyl acetoacetate, methyl salicylate [Loba Chemie], hydrazine hydrate [Fisher Scientific], ammonium molybdate tetrahydrate, cadmium(II) acetate dihydrate, zinc(II) acetate dihydrate, hexadecaaquaoctahydroxotetrazirconium(IV) chloride [BDH], dioxouranium(VI) acetate dihydrate [Hopkins and Williams (UK)], DMSO, DMF, MeOH, EtOH, 1,4-dioxane, and THF [Ranbaxy] were used as received for the syntheses. Bis(acetylacetonato)dioxomolybdenum(VI) and hexadecaaquaoctahydroxotetrazirconium(IV) acetate were synthesized according to the literature procedures [30, 31]. All the microbial cultures were procured from microbial type culture collection (MTCC), IMTECH, Chandigarh. The bacteria were subcultured on nutrient agar, whereas yeast was subcultured on malt yeast agar. 

### 2.2. Analytical and Physical Measurements

The estimation of metal contents, spectral studies (IR, reflectance, ^1^H NMR, ESR), and the magnetic susceptibility measurements were carried out by the methods reported earlier [32]. The melting points of the compounds were determined on digital melting point apparatus (Stuart SMP-40). For the purification of KBHz, SHz and **1–7** chromatographic separations were carried out using silica gel columns (160–200 mesh) of varying length. Thin-layer chromatography (TLC) was performed on commercial Merck plates coated with a 0.20 mm layer of silica gel. The molar conductances of the coordination compounds in DMSO were carried out using Toshniwal conductivity bridge (Model CL01-02A) and a dip type cell calibrated with KCl solution. Carbon, hydrogen, and nitrogen contents of the compounds were determined on a FLASH EA 1112 CHNS (O) analyzer. The IR spectra of **1**–**7** were recorded in KBr (4000–250 cm^−1^) on a Fourier Transform Infrared spectrometer (Model RZX, Perkin Elmer). The reflectance spectra were recorded on a Hitachi-330 UV-vis-NIR spectrophotometer. ^1^H NMR spectra of 3-ketobutanehydrazide, **1**, **4**–**7** were recorded on an Avance-II (Bruker) FT NMR spectrometer at 400 MHz using DMSO as a solvent and TMS as an internal standard. The mass spectrum of **1 **was recorded on Waters Micromass Q-Tof Micro-mass spectrometer. The ESR spectrum of **3** was recorded at LNT in solid on a Varian E-112 ESR spectrometer with *X*-band microwave frequency (9.1 GHz) using tetracyanoethylene (TCNE) as a *g*-marker and monitoring the frequency with a frequency meter. The magnetic measurements were carried out at room temperature by Lakeshore VSM 7410 instrument. The antimicrobial studies of **1**–**7** were performed by agar well diffusion method [33–35].

### 2.3. Antibacterial Activity

A total of six microbial strains, that is, two gram-positive bacteria (*S*. *aureus*, *B*.* subtilis*), two gram-negative bacteria (*E*.* coli*, *P*.* aeruginosa*), and two yeasts (*S*. *cerevisiae*, *C*.* albicans*), were screened for evaluation of antibacterial and antifungal activities of **1**–**7**. All the microbial cultures were adjusted to 0.5 McFarland standard, which were visually comparable to a microbial suspension of approximately 1.5 × 10^8^ cfu/mL. 20 mL of agar medium was poured into each Petri plate, and the agar plates were swabbed with 100 *μ*L inocula of each test microorganism and kept for 15 min for adsorption. Using sterile cork borer of 8 mm diameter, wells were bored into the seeded agar plates and these were loaded with a 100 *μ*L volume with concentration of 2.0 mg/mL of each compound reconstituted in DMSO. All the plates were incubated at 37°C for 24 h. The antimicrobial activity of each compound was evaluated by measuring the zone of growth inhibition against the test microorganisms with zone reader (Hi antibiotic zone scale). DMSO was used as a negative control, whereas ciprofloxacin and amphotericin B were used as positive controls for bacteria and yeasts, respectively.

#### 2.3.1. Determination of Minimum Inhibitory Concentration (MIC)

The minimum inhibitory concentration (MIC) is the lowest concentration of an antimicrobial compound that inhibits the visible growth of a microorganism after overnight incubation. MIC of the various compounds against bacterial and yeast strains was tested through a modified agar well diffusion method [36]. In this method, a two-fold serial dilution of each compound was prepared by first reconstituting the compound in DMSO followed by dilution in sterile distilled water to achieve a decreasing concentration range of 512 to 1 *μ*g/mL. 100 *μ*L of each dilution was introduced into wells (in triplicate) in the agar plates already seeded with 100 *μ*L of standardized inoculums (10^6^ cfu/mL) of the test microbial strain. All test plates were incubated aerobically at 37°C for 24 h, and the inhibition zones were observed. MIC was recorded for each test organism.

### 2.4. Synthesis and Characterization

#### 2.4.1. Synthesis of 3-Ketobutanehydrazide (KBHz)

Hydrazine hydrate (5.0 g, 100 mmol) was added slowly with continuous stirring to an ice-cooled EtOH solution (20 mL) of ethyl acetoacetate (13.0 g, 100 mmol) during a period of 0.5 h. The reaction mixture was refluxed on a water bath for 2 h. The white compound separated out was suction filtered, washed with EtOH and recrystallised from EtOH, and dried *in vacuo* over silica gel at room temperature. The progress of the reaction was monitored on TLC using hexane and Et_2_O (1 : 1 v/v) as eluent. Color: white. M. p. = 188°C. Yield: 10.4 g (90%). Anal. Calcd. for C_4_H_8_N_2_O_2_: C, 41.38; H, 6.90; N, 24.06; Found: C, 41.24; H, 6.94; N, 24.14. IR bands (cm^−1^): 3298 *ν*(OH) (intramolecular H-bond), 2899 *ν*(N–H) (intramolecular H-bond), 1677 *ν*(C=O) (keto), 1618 *δ*(NH_2_), and 1041 *ν*(N–N) (hydrazide). ^1^H NMR (400 MHz; DMSO-d_6_; **δ**, ppm): 1.27 (s, 3H, –CH_3_), 2.58 (s, 2H, –CH_2_), 5.26 (br, 2H, –NH_2_) and 7.84 (br, 1H, –CONH). 

#### 2.4.2. Synthesis of Salicylhydrazide (SHz)

The title compound was synthesized according to the literature procedure [37]. The progress of the reaction was monitored on TLC using hexane and Et_2_O (1 : 1 v/v) as eluent. Color: white shining crystals. M. p. = 147°C. Yield: 11.4 g (75%). Anal. Calcd. for C_7_H_8_N_2_O_2_: C, 55.26; H, 5.26; N, 18.42; Found: C, 55.29; H, 5.21; N, 18.43. IR bands (cm^−1^): 3434 *ν*(OH) (intramolecular H-bond), 3320 *ν*(N–H) (intramolecular H-bond), 1735 *ν*(C=O) (keto), 1643 *ν*(C=N), 1607 *δ*(NH_2_), 1532 *ν*(C–O) (*ϕ*), 1252 *ν*(C–O) (enolic), 1035 *ν*(N–N) (hydrazide). ^1^H NMR (400 MHz; DMSO-d_6_; **δ**, ppm): 4.18 (s, 2H, –NH_2_), 6.80–8.04 (m, 4H, ArH), 9.90 (br, 1H, phenolic-OH) and 12.33 (br, 1H, enolic-OH).

#### 2.4.3. Synthesis of **1**

3-Ketobutanehydrazide (11.6 g, 100 mmol) and salicylhydrazide (15.2 g, 100 mmol) were refluxed in EtOH (50 mL) on a water bath for 2 h. The excess of solvent was distilled off, and the yellow compound separated out was allowed to stand at room temperature. The compound was suction filtered, washed with EtOH and recrystallized from EtOH, and dried as mentioned above. The progress of the reaction was monitored on TLC using hexane and Et_2_O (1 : 1 v/v) as eluent. Color: yellow. M. p. = 109°C. Yield: 22.5 g (90%). Anal. Calcd. for C_11_H_14_N_4_O_3_: C, 52.80; H, 5.60; N, 22.36; Found: C, 52.69; H, 5.71; N, 22.40. IR bands (cm^−1^): 3267 *ν*(OH) (intramolecular H-bond), 2720 *ν*(N–H) (intramolecular H-bond), 1619 *ν*(C=N) (azomethine), 1532 *ν*(C–O)*ϕ*, 1239 *ν*(C–O) (enol) and 1012 *ν*(N–N).^ 1^H NMR (400 MHz; DMSO-d_6_; **δ**, ppm): 2.14 (s, 3H, –CH_3_), 2.56 (s, 2H, –CH_2_), 5.24 (d, 2H, –NH_2_), 6.84–7.80 (m, 4H, –ArH), 8.01 (s, 1H, –N=COH) (adjacent to aliphatic moiety), 9.87 (br, 1H, –OH) (phenolic), 12.24 (s, 1H, –N=COH) (adjacent to aromatic moiety).

#### 2.4.4. Syntheses of ** 2–7**

A MeOH solution (~30 mL) of appropriate metal acetate (5 mmol) was added to a MeOH solution (~100 mL) of **1 **(1.25 g, 5 mmol) with constant stirring. The solution was refluxed on a water bath for 3-4 h, and the solid residue obtained was suction filtered, washed with MeOH, and dried as mentioned above. The resulting solids were recrystallized from dimethyl sulfoxide (DMSO). The progress of the reaction was monitored on TLC using hexane and Et_2_O (1 : 1 v/v) as eluent. Color: mentioned in Table 1. Yield: 50–75%. The compounds are stable up to 250°C, and they get decomposed above this temperature. ^1^H NMR spectral data of these coordination compounds are given in Table 3. We were unable to get the compounds (**1–7**) in crystalline forms; therefore, their studies related with X-ray structural determinations could not be carried out.

## 3. Results and Discussion 

**Scheme 1:**
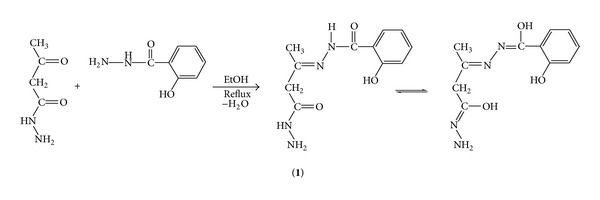
Synthesis of the Schiff base.

The nucleophilic addition reaction between 3-ketobutanehydrazide and salicylhydrazide in equimolar ratio in EtOH followed by the elimination of one water molecule results in the formation of the Schiff base, LH_3_ (**1**) (Scheme 1). 

**Scheme 2:**
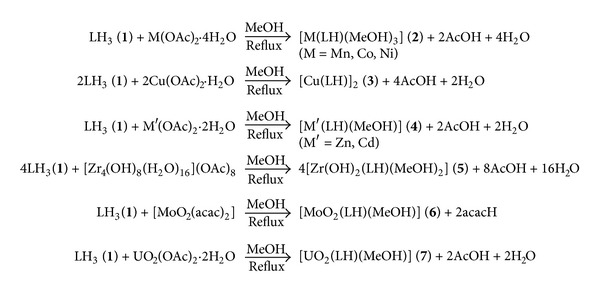
Synthesis of complexes **2**-**7**.

A MeOH solution of **1 **reacts with a MeOH solution of Mn(II), Co(II), Ni(II), Cu(II), Zn(II), Cd(II), Zr(OH)_2_(IV), MoO_2_(VI), and UO_2_(VI) ions and forms the corresponding coordination compounds, **2**–**7** (Scheme 2).

The coordination compounds are insoluble in H_2_O, EtOH, dioxane, and THF, but they were soluble in DMF and DMSO. Their molar conductance data (3.6–11.7 Ω^−1^ cm^2^ mol^−1^ in DMSO) reveal their nonelectrolytic nature. They are stable up to 250°C and get decomposed above this temperature. Attempts to obtain single crystal suitable for X-ray determination were unsuccessful. The structures of the synthesized ligand and metal complexes (Schemes 1 and 2) were established with the help of elemental analyses data, IR and NMR spectra.

### 3.1. Infrared Spectral Studies

The IR spectra of KBHz and **1**–**7** were recorded in KBr. The *ν*(C=N) (azomethine) stretch of **1** shifts to lower energy by 7–24 cm^−1^ indicating coordination through its azomethine N atom [38]. The *ν*(C–O)*ϕ* stretch of **1** occurring at 1532 cm^−1^ remains unaltered in **2**–**7** indicating the noninvolvement of phenolic O atom towards coordination [39]. The *ν*(C–O) (enolic) stretch of the Schiff base shifts from 1239 cm^−1^ to higher energy by 8–18 cm^−1^ in **2–7** indicating coordination through its enolic O atom [39]. Thus, **1** behaves as a dibasic tridentate ONO donor ligand in **2**–**7 **coordinating through its azomethine N and both enolic O atoms. The involvement of enolic O and azomethine N atoms towards coordination is further supported by the appearance of new nonligand bands between 571–594 and 478–483 cm^−1^ due to the *ν*(M–O) and *ν*(M–N) vibrations in **2**–**7**. These bands are in the expected order of increasing energy: *ν*(M–N) < *ν*(M–O) [40] as expected due to the greater dipole moment change in the M–O vibration, greater electronegativity of the O atom than N atom, and shorter M–O bond length than the M–N bond length [41]. The absence of a band, between 835–955 cm^−1^, characteristic of the *ν*(Zr=O) stretch [42] and the appearance of a new band at 1125 cm^−1^ due to *δ*(Zr–OH) bending mode in **5 **suggests the structure of **5** structure as [Zr(OH)_2_(LH)(MeOH)_2_] and not as [ZrO(H_2_O)(LH)(MeOH)_2_]. The *ν*_s_(O=Mo=O) and *ν*_as_(O=Mo=O) stretches occur at 925 and 900 cm^−1^, respectively in **6**, and these bands occur in the usual range (892–964 cm^−1^; 840–925 cm^−1^) reported for the majority of MoO_2_(VI) compounds [43]. The presence of the *ν*_s_(O=Mo=O) and *ν*_as_(O=Mo=O) bands indicates a *cis-*MoO_2_ structure as the compounds with a *trans-*MoO_2_ structure exhibit only the *ν*_as_(O=Mo=O) stretch since the *ν*_s_(O=Mo=O) stretch is IR inactive [44]. The absence of a band at ~770 cm^−1^ in the present MoO_2_(VI) coordination compound indicates the absence of an oligomeric structure with *⋯*Mo=O*⋯*Mo=O*⋯* interaction [43]. The *ν*_as_(O=U=O) stretch in **7** occurs at 930 cm^−1^. This band occurs in the usual range (870–950 cm^−1^) observed for the majority of *trans*-UO_2_ compounds [45].

### 3.2. Reflectance Spectral Studies

The coordination compound **2** (M = Mn) shows three bands at 15860, 21275, and 25850 cm^−1^ due to the ^6^ *A*_1g_ → ^4^ *T*_1g_(*G*)(*ν*_1_), ^6^ *A*_1g_ → ^4^ *T*_2g_(*G*)(*ν*_2_) and ^6^ *A*_1g_ → ^4^ *A*_1g_(*G*) (*ν*_3_) transitions, respectively, in an octahedral environment [46]. The coordination compound **2** (M = Co) shows three bands at 9091, 13698 and 19820 cm^−1^ due to the ^4^ *T*_1g_(*F*) → ^4^ *T*_2g_(*F*)(*ν*_1_), ^4^ *T*_1g_(*F*) → ^4^ *A*_2g_(*F*)(*ν*_2_) and ^4^ *T*_1g_(*F*) →^ 4^ *T*_1g_(*P*)(*ν*_3_) transitions, respectively, in an octahedral environment [46]. Using the free ion value of *B* = 971 cm^−1^, the values of spectral parameters [46] in **2** (M = Co) are as follows: 10*Dq* = 10253 cm^−1^, *B*′ = 792.74 cm^−1^, **β** = *B*′/*B* = 0.82, **β** ^0^ = 18% and CFSE = –98.20 kJ mol^−1^. The value of *ν*_3_/*ν*_1_ is 2.18, and this value falls in the usual range (2.00–2.80) observed for the majority of octahedral Co(II) coordination compounds [46]. The coordination compound **2** (M = Ni) shows three bands at 9250, 15360, and 24095 cm^−1^ due to the ^3^ *A*_2g_(*F*) → ^3^ *T*_2g_(*F*)(*ν*_1_), ^3^ *A*_2g_(*F*) → ^3^ *T*_1g_(*F*)(*ν*_2_) and ^3^ *A*_2g_(*F*) → ^3^ *T*_1g_(*P*)(*ν*_3_) transitions, respectively, suggesting an octahedral geometry around the metal ion [46]. Using the free ion value of *B* = 1030 cm^−1^, the values of spectral parameters in **2** (M = Ni) are as follows: 10*Dq *= 9250 cm^−1^, *B*′ = 743.74 cm^−1^, **β** = 0.72, **β** ^0^ = 28%, and CFSE = –132.79 kJ mol^−1^. The value of the *ν*_2_/*ν*_1_ is 1.66, and this value lies in the usual range (1.6–1.8) reported for the majority of octahedral Ni(II) coordination compounds [46]. The 10*Dq* value of the Co(II) coordination compound is greater than that of the corresponding Ni(II) coordination compound 10253 cm^−1^ > 9250 cm^−1^. This is in line with the spectrochemical series of metal ions for a given ligand, given stoichiometry, and a given stereochemistry: Co(II) > Ni(II) [46]. The **β** ^0^ value of the Co(II) coordination compound is less as compared to that of the corresponding Ni(II) coordination compound: 18% < 28%. This is in line with the nephelauxetic metal ion series in terms of **β** and **β** ^0^ for a given ligand, a given stoichiometry, and a given stereochemistry [46]. The coordination compound **3** shows two bands: one at 14750 cm^−1^ and the other at 20150 cm^−1^ due to the ^2^ *B*_1g_ → ^2^ *A*_1g_ and ^2^ *B*_1g_ → ^2^ *E*_g_ transitions, respectively, indicating a square-planar configuration around the metal ion [46] (Table 2).

### 3.3. ^1^H NMR Studies

The ^1^H NMR spectra of KBHz, **1** and **4–7** were recorded in DMSO-d_6_. The chemical shifts (**δ**) are expressed in ppm downfield from TMS [47]. The Schiff base (**1**) exhibits a singlet at *δ* 2.14 ppm due to the methyl protons, a singlet at *δ* 2.56 ppm due to the methylene proton, a doublet at *δ* 5.24 ppm due to the –NH_2_ protons, a broad signal at *δ* 9.87 ppm due to the phenolic proton, a multiplet at **δ** 6.84–7.80 ppm due to the aromatic protons, a singlet at **δ** 8.01 ppm due to –N=COH (adjacent to aliphatic moiety) proton, and a singlet at *δ* 12.24 ppm due to –N=COH (adjacent to aromatic moiety) proton. The absence of the resonance signals at *δ* 8.01 ppm and *δ* 12.24 ppm due to the enolic protons (adjacent to aliphatic and aromatic moieties, resp.) in **4**–**7** indicates the deprotonation of the enolic protons followed by the involvement of both enolic O atoms towards coordination. 

### 3.4. ESR Spectral Studies

The ESR spectrum of **3 **in DMSO at liquid nitrogen temperature was recorded in *X*-band, using 100 kHz field modulation, and the *g* values are relative to the standard marker, tetracyanoethylene (TCNE) (*g* = 2.0023). The observed values of *g*_||_, *g*_⊥_, *g*_av_, and *G* are 2.15, 2.08, 2.11, and 1.84, respectively. From the observed values of various parameters, it is concluded that the unpaired electron lies in *d*_*x*_^2^ − *y* ^2^ orbital giving ^2^ *B*_1_ as the ground state with *g*_||_ > *g*_⊥_ > 2, indicating square planar geometry around the copper(II) ion [48, 49].

### 3.5. Magnetic Measurements

The magnetic moments of **2** (M = Mn, Co, Ni) are 5.86, 4.78, and 3.17 B.M., respectively. These values lie in the normal ranges reported for the majority of magnetically dilute octahedral compounds of Mn(II), Co(II), and Ni(II) ions [46]. The magnetic moment of **3** (M = Cu) is 1.76 B.M. indicating square planar geometry around the Cu(II) ion [46]. The coordination compounds **4–7** are diamagnetic. 

### 3.6. Antimicrobial Studies

**Figure 1:**
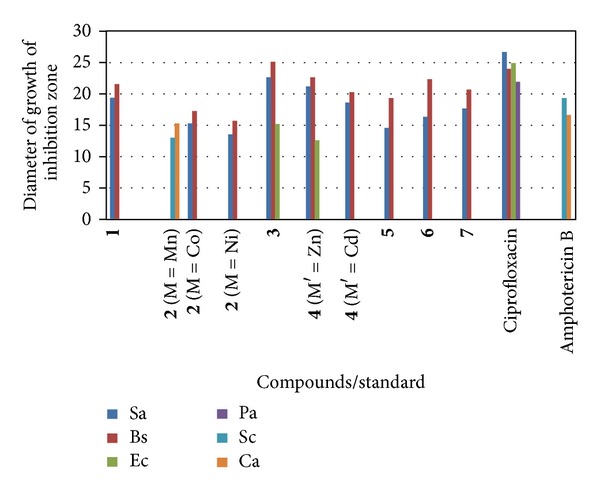
Bar Chart indicating the diameter of growth of inhibition zone for compounds/standard against various microbes.

**Figure 2:**
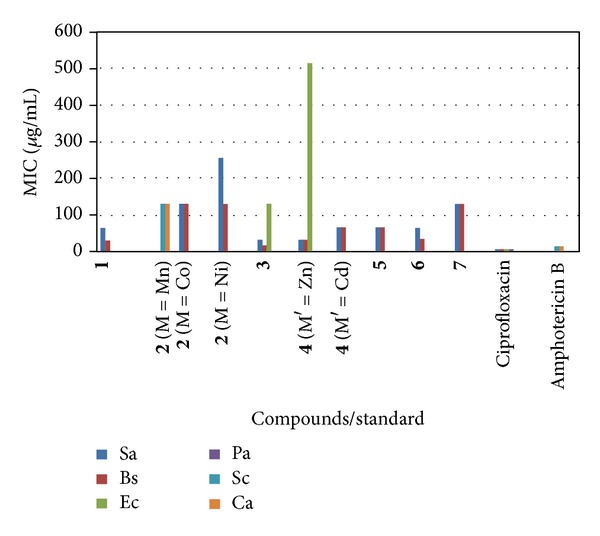
Bar Chart indicating minimum inhibitory concentration (MIC) (*μ*g/mL) for compounds/standard against various microbes. Abbreviations: Sa: *S. aureus*, Bs: *B. subtilis*, Ec: *E. coli*, Pa: *P. aeruginosa*, Sc: *S. cerevisiae*, Ca: *C. albicans. *

The newly synthesized compounds (**1**–**7**) were screened for their antibacterial and antifungal activities (Tables 4 and 5). The compounds **1, 2** (M = Co, Ni) and** 3–7 **possessed variable antibacterial activities against the gram-positive bacteria (*S*.* aureus*, *B*. *subtilis*). The compounds **3** and **4** (M′  = Zn) displayed activities against gram-negative bacteria (*E. coli*). The compound **2 **(M = Mn) displayed antifungal activities against yeasts (*S*.* cerevisiae*, *C*.* albicans*). Positive controls produced significantly sized inhibition zones against the tested bacteria and fungi; however, negative control produced no observable inhibitory effect against any of the test organisms (Figures 1 and 2). On the basis of maximum inhibitory activities shown against gram-positive bacteria, the compounds **3** and **4** (M′  = Zn) were found to be most effective against *S. aureus* with zone of inhibition of 22.6 mm and 21.3 mm. The compound **3** was found to be most effective against *B. subtilis *showing the zone of inhibition of 25.3 mm. Among gram-negative bacteria, the compounds **3** and **4** (M′  = Zn) displayed antibacterial activities with zone of inhibition of 15.3 mm and 12.6 mm against *E. coli.* The compound **2** (M = Mn) showed zone of inhibition ranging between 13.0 mm against *S*.* cerevisiae* and 15.3 mm against *C*.* albicans*. The MIC of various compounds ranged between 16 *μ*g/mL and 256 *μ*g/mL against gram-positive bacteria, while it ranged between 128 *μ*g/mL and 512 *μ*g/mL against gram-negative bacteria. The compounds **3** and **4 **(M′ = Zn) were found to be the best as they exhibit the lowest MIC of 32 *μ*g/mL against* S. aureus*. The compound **3 **showed lowest MIC of 16 *μ*g/mL against *B. subtilis*. However, in case of yeasts, the compound **2** (M = Mn) showed MIC value of 128 *μ*g/mL. The compound **3 **was found to be the best in inhibiting the growth of bacteria; thus, it can be further used as an antibacterial agent in pharmaceutical industry for mankind, after testing its toxicity to human beings. It is worth to mention that the antimicrobial activity of the ligand (**1**) is greatly enhanced after coordination [50–52]. The lipid membrane surrounding the cell favours the passage of only lipid-soluble materials; therefore, the liposolubility is an important factor which controls the antimicrobial activity [53, 54]. On chelation, the polarity of the metal ion is reduced to a greater extent due the overlapping of the ligand orbital and partial sharing of the positive charge of the metal ion with donor groups. Moreover, delocalization of the **π**-electrons over the whole chelate ring is increased and the lipophilicity of the coordination compounds is enhanced. The increased lipophilicity enhances the penetration of the coordination compounds into the lipid membranes and blocks the metal binding sites in the enzymes of microorganisms. These coordination compounds also disturb the respiration process of the cell and thus block the synthesis of proteins, which restricts further growth of the microorganisms. In general, coordination compounds are more active than ligand.

## 4. Conclusions

**Scheme 3:**
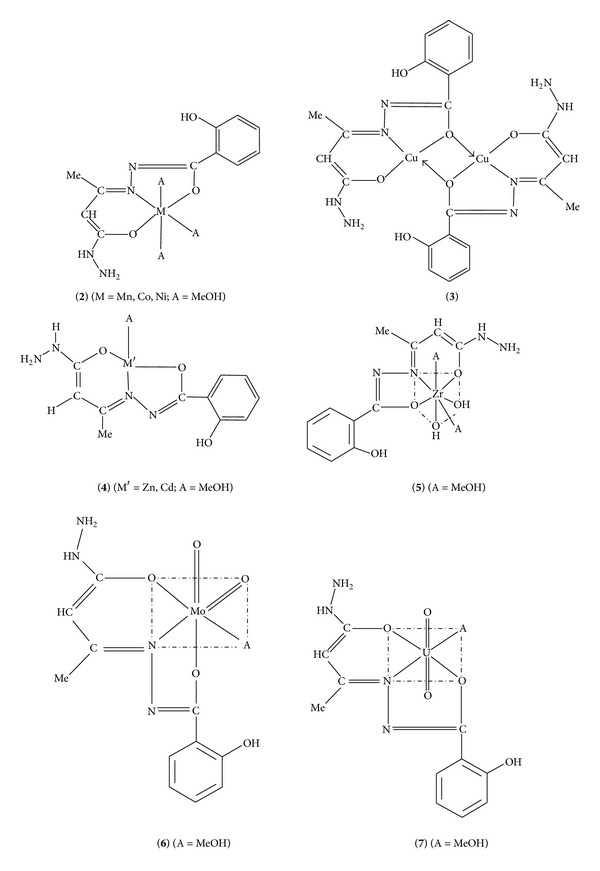


On the basis of the analytical data, valence requirements, conductance, spectral studies, and magnetic susceptibility measurements, it is proposed that **1** acts as a monobasic tridentate ONO donor ligand in **2–7 **coordinating through its azomethine N and both enolic O atoms. **2 **and** 3 **are paramagnetic, while **4–7** are diamagnetic. The data suggest a square-planar structure to **3**, a tetrahedral structure to **4**, an octahedral structure to **2**, **6**, and **7**, and a pentagonal bipyramidal structure to** 5**. The coordination compounds show significant enhanced antimicrobial activities as compared to the free Schiff base (Scheme 3). Therefore, these compounds can be further used in pharmaceutical industry as antimicrobial agents for mankind, after testing its toxicity to human beings.

## Figures and Tables

**Table 1:** Analytical, colour, molar conductance (Ω^−1^ cm^2^ mol^−1^), mass spectral, and molecular weight data of compounds.

S. no.	Compound	Stoichiometry	Colour	Yield g (%)	Λ_M_ (Ω^−1^ cm^2^ mol^−1^)	Found (calcd)				
						M. Wt.	M%	C%	H%	N%
(1)	**1**	C_11_H_14_N_4_O_3_	Yellow	22.5 (90)	—	250.1^a^ (250)	—	52.69 (52.80)	5.71 (5.60)	22.36 (22.40)
(2)	**2** (M = Mn)	MnC_14_H_24_N_4_O_6_	Grey	1.14 (57)	11.7	352.6^b^ (398.9)	13.63 (13.76)	42.08 (42.12)	6.11 (6.02)	14.17 (14.04)
(3)	**2** (M = Co)	CoC_14_H_24_N_4_O_6_	Brown	1.25 (62)	10.3	423.4^b^ (402.9)	14.43 (14.62)	41.82 (41.70)	5.88 (5.96)	13.72 (13.90)
(4)	**2** (M = Ni)	NiC_14_H_24_N_4_O_6_	Purple	1.37 (68)	9.2	392.6^b^ (402.7)	14.67 (14.58)	41.79 (41.72)	5.87 (5.96)	13.73 (13.91)
(5)	**3**	Cu_2_C_22_H_24_N_8_O_6_	Dark Green	1.12 (72)	6.8	618.4^b^ (623.0)	20.52 (20.39)	42.46 (42.38)	3.73 (3.85)	17.82 (17.98)
(6)	**4** (M′ = Zn)	ZnC_12_H_16_N_4_O_4_	Yellow	0.86 (50)	5.6	363.9^b^ (345.4)	18.65 (18.93)	41.52 (41.69)	4.73 (4.63)	16.08 (16.21)
(7)	**4** (M′ = Cd)	CdC_12_H_16_N_4_O_4_	White	1.18 (60)	5.2	387.3^b^ (392.4)	28.52 (28.64)	36.82 (36.70)	4.13 (4.08)	14.31 (14.27)
(8)	**5**	ZrC_13_H_22_N_4_O_7_	Yellow	1.64 (75)	5.0	425.8^b^ (437.2)	20.93 (20.86)	35.63 (35.68)	5.08 (5.03)	12.72 (12.81)
(9)	**6**	MoC_12_H_16_N_4_O_6_	Yellow	1.18 (58)	4.7	419.6^b^ (407.9)	23.48 (23.51)	35.23 (35.30)	3.84 (3.92)	13.52 (13.73)
(10)	**7**	UC_12_H_16_N_4_O_6_	Orange	1.79 (65)	3.6	541.7^b^ (550.0)	43.44 (43.27)	26.36 (26.18)	2.88 (2.91)	10.02 (10.18)

Abbreviations: ^a^mass spectral data and ^b^Rast method data.

**Table 2:** IR, reflectance spectral data (cm^−1^), and magnetic moments of the coordination compounds.

S. no.	Compound	*ν* (C=N) (azomethine)	*ν* (C–O) (enolic)	*ν*_max⁡_ (cm^−1^)	Magnetic moment (B.M.)
(1)	**1**	1619	1239	—	—
(2)	**2** (M = Mn)	1604	1256	15860, 21275, 25850	5.86
(3)	**2** (M = Co)	1601	1247	9091, 13698, 19820	4.78
(4)	**2** (M = Ni)	1605	1257	9250, 15360, 24095	3.17
(5)	**3 **	1605	1248	14750, 20150	1.76
(6)	**4** (M′ = Zn)	1606	1257	—	Diamagnetic
(7)	**4** (M′ = Cd)	1608	1254	—	Diamagnetic
(8)	**5**	1612	1250	—	Diamagnetic
(9)	**6**	1598	1252	—	Diamagnetic
(10)	**7**	1595	1248	—	Diamagnetic

**Table 3:** NMR spectral data of the coordination compounds.

S. no.	Compound	Stoichiometry	^ 1^H NMR (400 MHz; DMSO-d_6_) **δ** (ppm)
(1)	**4 **(M′ = Zn)	ZnC_12_H_16_N_4_O_4_	1.27 (t, 3H, –CH_3_), 2.06 (s, 3H, –CH_3_) (MeOH), 2.56 (s, 1H, –CH), 3.53 (br, 1H, –OH) (MeOH), 5.14 (d, 2H, –NH_2_), 6.94–7.80 (m, 4H, Ar–H), 8.50 (br, 1H, –NH), 9.87 (br, 1H, –OH) (phenolic)
(2)	**4** (M′ = Cd)	CdC_12_H_16_N_4_O_4_	1.25 (t, 3H, –CH_3_), 2.15 (s, 3H, –CH_3_) (MeOH), 2.58 (s, 1H, –CH), 3.16 (br, 1H, –OH) (MeOH), 5.14 (d, 2H, –NH_2_), 6.74–7.88 (m, 4H, Ar–H), 8.56 (br, 1H, –NH), 9.87 (br, 1H, –OH) (phenolic)
(3)	**5**	ZrC_13_H_22_N_4_O_7_	1.30 (t, 3H, –CH_3_), 2.15 (s, 3H, –CH_3_) (MeOH), 2.50 (s, 3H, –CH_3_) (MeOH), 2.58 (s, 1H, –CH), 3.16 (br, 2H, –OH), 3.26 (br, 2H, –OH) (MeOH), 5.14 (d, 2H, –NH_2_), 6.74–7.88 (m, 4H, Ar–H), 8.72 (br, 1H, –NH), 9.87 (br, 1H, –OH) (phenolic)
(4)	**6**	MoC_12_H_16_N_4_O_6_	1.25 (t, 3H, –CH_3_), 2.25 (s, 3H, –CH_3_) (MeOH), 2.56 (s, 1H, –CH), 3.45 (br, 1H, –OH) (MeOH), 5.11 (d, 2H, –NH_2_), 6.53–7.50 (m, 4H, Ar–H), 8.87 (br, 1H, –CONH), 9.90 (br, 1H, –OH) (phenolic)
(5)	**7**	UC_12_H_16_N_4_O_6_	1.25 (t, 3H, –CH_3_), 2.35 (s, 3H, –CH_3_) (MeOH), 2.56 (s, 1H, –CH), 3.52 (br, 1H, –OH) (MeOH), 5.14 (d, 2H, –NH_2_), 6.66–7.52 (m, 4H, Ar–H), 8.87 (br, 1H, –CONH), 9.90 (br, 1H, –OH) (phenolic)

**Table 4:** *In  vitro* antimicrobial activity of synthetic chemical compounds through agar well diffusion method.

Compound no.	Diameter of growth of inhibition zone (mm)^a^					
	*S*.* aureus *	*B*.* subtilis *	*E*.* coli *	*P*.* aeruginosa *	*S*.* cerevisiae *	*C*.* albicans *
**1**	19.3	21.6	—	—	—	—
**2 **(M = Mn)	—	—	—	—	13.0	15.3
**2 **(M = Co)	15.3	17.3	—	—	—	—
**2** (M = Ni)	13.6	15.6	—	—	—	—
**3**	22.6	25.3	15.3	—	—	—
**4 **(M′ = Zn)	21.3	22.6	12.6	—	—	—
**4 **(M′ = Cd)	18.6	20.3	—	—	—	—
**5**	14.6	19.3	—	—	—	—
**6**	16.3	22.3	—	—	—	—
**7**	17.6	20.6	—	—	—	—
Ciprofloxacin	26.6	24.0	25.0	22.0	—	—
Amphotericin B	—	—	—	—	19.3	16.6

—: no activity, ^a^values, including diameter of the well (8 mm), are means of three replicates.

**Table 5:** Minimum inhibitory concentration (MIC) (*µ*g/mL) of compounds by using modified agar well diffusion method.

Compound no.	*S*.* aureus *	*B*.* subtilis *	*E*.* coli *	*P. aeruginosa *	*S*.* cerevisiae *	*C*.* albicans *
**1**	64	32	—	—	—	—
**2 **(M = Mn)	—	—	—	—	128	128
**2 **(M = Co)	128	128	—	—	—	—
**2** (M = Ni)	256	128	—	—	—	—
**3**	32	16	128	—	—	—
**4 **(M′ = Zn)	32	32	512	—	—	—
**4 **(M′ = Cd)	64	64	—	—	—	—
**5**	64	64	—	—	—	—
**6**	64	32	—	—	—	—
**7**	128	128	—	—	—	—
Ciprofloxacin	6.25	6.25	6.25	6.25	—	—
Amphotericin B	—	—	—	—	12.5	12.5

—: no activity.

## References

[1] Shah S, Vyas R, Mehta RH (1992). Synthesis, characterization and antibacterial activities of some new Schiff base compounds. *Journal of Indian Chemical Society*.

[2] Pandeya SN, Sriram D, Nath G, Clercq ED (1999). Synthesis, antibacterial, antifungal and anti-HIV activities of Schiff and Mannich bases derived from isatin derivatives and N-[4-(4′-chlorophenyl)thiazol-2-yl] thiosemicarbazide. *European Journal of Pharmaceutical Sciences*.

[3] More PG, Bhavankar RB, Patter SC (2001). Synthesis and biological activity of Schiff bases of aminothiazoles. *Journal of Indian Chemical Society*.

[4] Leite ACL, de Lima RS, Moreira DR (2006). Synthesis, docking and *in vitro* activity of thiosemicarbazones, aminoacyl-thiosemicarbazides and acyl-thiazolidones against *Trypanosoma cruzi*. *Bioorganic Medicinal Chemistry*.

[5] Smalley TL, Peat AJ, Boucheron JA (2006). Synthesis and evaluation of novel heterocyclic inhibitors of GSK-3. *Bioorganic Medicinal Chemistry Letters*.

[6] Gemma S, Kukreja G, Fattorusso C (2006). Synthesis of *N*1-arylidene-*N*2-quinolyl- and *N*2-acrydinylhydrazones as potent antimalarial agents active against CQ-resistant *P. falciparum* strains. *Bioorganic Medicinal Chemistry Letters*.

[7] Nayyar A, Monga V, Malde A, Coutinho E, Jain R (2007). Synthesis, anti-tuberculosis activity and 3D-QSAR study of 4-(adamantan-1-yl)-2-substituted quinolines. *Bioorganic Medicinal Chemistry*.

[8] Hanna ML, Tarasow TM, Perkins J (2007). Mechanistic differences between *in vitro* assays for hydrazone-based small molecule inhibitors of anthrax lethal factor. *Bioorganic Medicinal Chemistry*.

[9] Visbal G, Marchan E, Maldonado A, Simoni Z, Navarro M (2008). Synthesis and characterization of platinum-sterol hydrazone complexes with biological activity against *Leishmania* (L.) *Mexicana*. *Journal of Inorganic Biochemistry*.

[10] Kumar P, Narasimhan B, Sharma D, Judge V, Narang R (2009). Hansch analysis of substituted benzoic acid benzylidene/furan-2-yl-methylene hydrazides as antimicrobial agents. *European Journal of Medicinal Chemistry*.

[11] Kumar D, Judge V, Narang R (2010). Benzylidene/2-chlorobenzylidene hydrazides: synthesis, antimicrobial activity, QSAR studies and antiviral evaluation. *European Journal of Medicinal Chemistry*.

[12] Yaul GAR, Dhande VV, Bhadange SG, Aswar AS (2011). Synthesis, structural studies and biological activity of dioxomolybdenum(VI), dioxotungsten(VI), thorium(IV) and dioxouranium(VI) complexes with 2-hydroxy-5-methyl and 2-hydroxy-5-chloroacetophenone benzoylhydrazone. *Russian Journal of Inorganic Chemistry*.

[13] Martin DF, Janusonis GA, Martin BB (1961). Stabilities of bivalent metal complexes of some *β*-ketoimines. *Journal of American Chemical Society*.

[14] Bahnasawy RME, Tabl ASE, Shereafy EE, Kashar TI, Issa YM (1999). Mononuclear and binuclear copper(II) complexes of phenylhydrazoacetylacetone isonicotinoylhydrazone. *Polish Joural of Chemistry*.

[15] Campos A, Anacona JR, Vallette MMC (1999). Synthesis and IR study of a zinc(II) complex containing a tetradentate macrocyclic Schiff base ligand: antifungal properties. *Main Group Metal Chemistry*.

[16] Verma M, Pandeya SN, Singh KN, Stables JP (2004). Anticonvulsant activity of Schiff bases of isatin derivatives. *Acta Pharmaceutica*.

[17] Fouda AS, Badr GE, El-Haddad MN (2008). The inhibition of C-steel corrosion in H_3_PO_4_ solution by some furfural hydrazone derivatives. *Journal of the Korean Chemical Society*.

[18] Narang KK, Aggarwal A (1974). Salicylaldehyde salicylhydrazone complexes of some transition metal ions. *Inorganica Chimica Acta*.

[19] Syamal A, Kumar D (1982). Molybdenum complexes of bioinorganic interest: new dioxomolybdenum(VI) complexes of Schiff bases derived from salicylaldehydes and salicylhydrazide. *Transition Metal Chemistry*.

[20] Syamal A, Kumar D (1983). Spectral studies on new dioxouranium(VI) complexes of tridentate Schiff bases derived from salicylhydrazide & salicylaldehyde or substituted salicylaldehydes. *Indian Journal of Pure and Applied Physics*.

[21] Baligar RS, Revankar VK (2006). Coordination diversity of new mononucleating hydrazone in 3d metal complexes: synthesis, characterization and structural studies. *Journal of Serbian Chemical Society*.

[22] Yang QX, Gang LZ, Sheng LW, Liang ZH (2008). Synthesis, crystal structure and cytotoxic activity of a novel nickel(II) complex with Schiff base derived from salicylhydrazide. *Chinese Journal of Structural Chemistry*.

[23] Chowdhury DA, Uddin MN, Sarker MAH (2008). Synthesis and characterization of dioxouranium(VI) complexes of some aroylhydrazines and their Schiff bases with acetone. *Chiang Mai Journal of Science*.

[24] Luo W, Wang XT, Meng XG, Cheng GZ, Ji ZP (2009). Metal coordination architectures of N-acyl-salicylhydrazides: the effect of metal ions and steric repulsion of ligands to their structures of polynuclear metal complexes. *Polyhedron*.

[25] Kumar D, Gupta PK, Kumar A, Dass D, Syamal A (2011). Syntheses, spectroscopic and magnetic properties of polystyrene-anchored coordination compounds of tridentate ONO donor Schiff base. *Journal of Coordination Chemistry*.

[26] Shelke VA, Jadhav SM, Shankarwar SG, Munde AS, Chondhekar TK (2011). Synthesis, characterization, antibacterial and antifungal studies of some transition and rare earth metal complexes of N-benzylidene-2-hydroxybenzohydrazide. *Bulletin Chemical Society of Ethiopa*.

[27] Gerber TIA, Yumata NC, Betz R (2012). The reaction of salicylhydrazide with [ReOX_3_(PPh_3_)_2_]. Influence of X on product formation. *Inorganic Chemistry Communications*.

[28] Al-Mawsawi LQ, Dayam R, Taheri L, Witvrouw M, Debyser Z, Neamati N (2007). Discovery of novel non-cytotoxic salicylhydrazide containing HIV-1 integrase inhibitors. *Bioorganic and Medicinal Chemistry Letters*.

[29] Neamati N, Hong H, Owen JM (1998). Salicylhydrazine-containing inhibitors of HIV-1 integrase: implication for a selective chelation in the integrase active site. *Journal of Medicinal Chemistry*.

[30] Chen GJJ, McDonald JW, Newton WE (1976). Synthesis of Mo(IV) and Mo(V) complexes using oxo abstraction by phosphines. Mechanistic implications. *Inorganic Chemistry*.

[31] Kumar D, Pandey V, Gupta A (2011). Studies on the coordination compounds of thiazolidin-4-one derived from salicylaldehyde-*o*-hydroxyphenylurea. *International Journal of Chemical Sciences*.

[32] Kumar D, Syamal A, Gupta A, Pandey V, Rani M (2012). Coordination compounds of Schiff base containing urea moiety. *Journal of Indian Chemical Society*.

[33] Ahmad I, Beg AZ (2001). Antimicrobial and phytochemical studies on 45 Indian medicinal plants against multi-drug resistant human pathogens. *Journal of Ethnopharmacology*.

[34] Andrews JM (2001). Determination of minimum inhibitory concentrations. *Journal of Antimicrobial Chemotherapy*.

[35] Obaidi OHSA (2012). Synthesis, characterization and theoretical treatment of sandwich Schiff bases complexes derived from salicylaldehyde with some transition metals and study of its biological activity. *International Journal of Chemistry Research*.

[36] Aneja KR, Sharma C, Joshi R (2011). *In vitro* efficacy of amaltas (*Cassia fistula* L.) against the pathogens causing otitis externa. *Jundishapur Journal of Microbiology*.

[37] Syamal A, Kumar D (1981). New oxozirconium(IV) complexes with the Schiff bases derived from salicylaldehyde, substituted salicylaldehydes and salicylhydrazide. *Polish Journal of Chemistry*.

[38] Mishra AP, Purwar H, Jain RK (2012). Microwave synthesis, spectral, thermal and antimicrobial activities of Co(II), Ni(II) and Cu(II) metal complexes with Schiff base ligand. *Biointerface Research in Applied Chemistry*.

[39] Syamal A, Kale KS (1979). Magnetic properties of oxovanadium(IV) complexes of some *β*-diketones. *Indian Journal of Chemistry*.

[40] Ferraro JR (1971). *Low Frequency Vibrations of Inorganic and Coordination Compounds*.

[41] Kumar D, Syamal A, Gupta A, Rani M, Gupta PK (2010). Role of pH on the formation of the coordination compounds with the Schiff base derived from 3-formylsalicylic acid and 4-amino-2,3-dimethyl-1-phenyl-3- pyrazolin-5-one. *Journal of the Indian Chemical Society*.

[42] Fouda AS, Badr GE, El-Haddad MN (2008). The inhibition of C-steel corrosion in H_3_PO_4_ solution by some furfural hydrazone derivatives. *Journal of the Korean Chemical Society*.

[43] Syamal A, Maurya MR (1989). Coordination chemistry of Schiff base complexes of molybdenum. *Coordination Chemistry Reviews*.

[44] Johnson NP, Lock CJL, Wilkinson G (1964). Amine, phosphine, arsine, and stibine complexes of rhenium-(III), -(IV), and -(V). *Journal of the Chemical Society*.

[45] Syamal A (1987). Calculation of electronic spectral parameters(Dq, *β*, *β*°, *λ*) and covalence for octahedral nickel (II), octahedral cobalt (II) and tetrahedral cobalt (II) complexes. *Chemistry Education*.

[46] Kumar D, Syamal A, Jaipal, Gupta PK (2007). Coordination compounds of polystyrene-supported azo dye. *Journal of the Indian Chemical Society*.

[47] Silverstein RM, Bassler GC (1967). *Spectrometric Identification of Organic Compounds*.

[48] Singh OI, Damayanti M, Singh NR, Singh RKH, Mohapatra M, Kadam RM (2005). Synthesis, EPR and biological activities of bis(1-N-butylamidino-O-alkylurea)copper(II)chloride complexes: EPR evidence for binuclear complexes in frozen DMF solution. *Polyhedron*.

[49] Boraey HAE, Rahman RMA, Atia EM, Hilmy KH (2010). Spectroscopic, thermal and toxicity studies of some 2-amino-3-cyano-1, 5-diphenylpyrrole containing Schiff bases copper(II) complexes. *Central European Journal of Chemistry*.

[50] Singh K, Kumar Y, Puri P, Kumar M, Sharma C (2012). Cobalt, nickel, copper and zinc complexes with 1, 3-diphenyl-1H-pyrazole-4-carboxaldehyde Schiff bases: antimicrobial, spectroscopic, thermal and fluorescence studies. *European Journal of Medicinal Chemistry*.

[51] Singh K, Kumar Y, Puri P, Singh G (2012). Spectroscopic, thermal and antimicrobial studies of Co(II), Ni(II), Cu(II) and Zn(II) complexes derived from bidentate ligands containing N and S donor atoms. *Bioinorganic Chemistry and Applications*.

[52] Patil SA, Unki SN, Kulkarni AD, Naik VH, Badami PS (2011). Co(II), Ni(II) and Cu(II) complexes with coumarin-8-yl Schiff-bases: spectroscopic, *in vitro* antimicrobial, DNA cleavage and fluorescence studies. *Spectrochimica Acta A*.

[53] Raman N, Kulandaisamy A, Jeyasubramanian K (2002). Synthesis, structural characterization, redox and antimicrobial studies of Schiff base copper(II), nickel(II), cobalt(II), manganese(II), zinc(II) and oxovanadium(II) complexes derived from benzil and 2-aminobenzyl alcohol. *Polish Journal of Chemistry*.

[54] Dharmaraj N, Viswanathamurthi P, Natarajan K (2001). Ruthenium(II) complexes containing bidentate Schiff bases and their antifungal activity. *Transition Metal Chemistry*.

